# The Glucose Metabolic Pathway as A Potential Target for Therapeutics: Crucial Role of Glycosylation in Alzheimer’s Disease

**DOI:** 10.3390/ijms21207739

**Published:** 2020-10-19

**Authors:** Vidyasagar Naik Bukke, Rosanna Villani, Moola Archana, Agata Wawrzyniak, Krzysztof Balawender, Stanislaw Orkisz, Luca Ferraro, Gaetano Serviddio, Tommaso Cassano

**Affiliations:** 1 Department of Clinical and Experimental Medicine, University of Foggia, 71122 Foggia, Italy; vidyasagar.bukke@unifg.it; 2 Department of Medical and Surgical Sciences, University of Foggia, 71122 Foggia, Italy; rosanna.villani@unifg.it (R.V.); archana.moola@unifg.it (M.A.); gaetano.serviddio@unifg.it (G.S.); 3 Morphological Science Department of Human Anatomy, Medical Faculty University of Rzeszów, 35-036 Rzeszów, Poland; a17041962@gmail.com (A.W.); balawender82@gmail.com (K.B.); sorkisz@ur.edu.pl (S.O.); 4 Department of Life Sciences and Biotechnology, University of Ferrara, 44100 Ferrara, Italy; luca.ferraro@unife.it

**Keywords:** Alzheimer’s disease, neurodegeneration, post-translational modifications, glycans, glycosylation, phosphorylation

## Abstract

Glucose uptake in the brain decreases because of normal aging but this decline is accelerated in Alzheimer’s disease (AD) patients. In fact, positron emission tomography (PET) studies have shown that metabolic reductions in AD patients occur decades before the onset of symptoms, suggesting that metabolic deficits may be an upstream event in at least some late-onset cases. A decrease in availability of glucose content induces a considerable impairment/downregulation of glycosylation, which is an important post-translational modification. Glycosylation is an important and highly regulated mechanism of secondary protein processing within cells and it plays a crucial role in modulating stability of proteins, as carbohydrates are important in achieving the proper three-dimensional conformation of glycoproteins. Moreover, glycosylation acts as a metabolic sensor that links glucose metabolism to normal neuronal functioning. All the proteins involved in β-amyloid (Aβ) precursor protein metabolism have been identified as candidates of glycosylation highlighting the possibility that Aβ metabolism could be regulated by their glycosylation. Within this framework, the present review aims to summarize the current understanding on the role of glycosylation in the etiopathology of AD, emphasizing the idea that glucose metabolic pathway may represent an alternative therapeutic option for targeting AD. From this perspective, the pharmacological modulation of glycosylation levels may represent a ‘sweet approach’ to treat AD targeting new mechanisms independent of the amyloid cascade and with comparable impacts in familial and sporadic AD.

## 1. Introduction

Glycans are essential for life along with DNA, proteins and lipids. They are one of the four basic components of the cell and contribute significantly to all physiological processes [1]. Glycans are composed of 10–15 monosaccharide residues, which are connected by covalent glycosidic bonds to proteins and lipids forming glycoproteins and glycolipids respectively, and able to modulate their function [2]. Glycan structures on glycoproteins and glycolipids are crucial during neural development and are responsible for cell adhesion, signal transduction, molecular trafficking, and differentiation. Glycosylation not only determines the conformation of protein but also influences properties including solubility, antigenicity, half-life and subcellular location [3].

Glycosylation also acts as a cellular regulator of growth and proliferation. In fact, the alteration of the glycosylation pathway in mouse embryonic fibroblasts causes growth delays, increased cyclin inhibitor p27 levels, and cell death [4]. Glycosylation not only has an important role in many fundamental cellular processes, but also its dysregulation contributes to the aetiology of important human diseases, particularly diabetes and neurodegeneration [5,6,7]. As far as the nervous system, the importance of glycans is highlighted by congenital glycosylation diseases, which result in psychomotor difficulties, mental retardation and other neuropathological symptoms. Nearly 30 such genetic disorders have been identified in the past decade, affecting the glycosylation pathways [8]. Therefore, understanding the functional roles of glycans in the nervous system will undoubtedly add another dimension to our knowledge of nervous system function, and glycans might become new targets for interventions to treat pathological conditions.

Glycans are very complex biological molecules composed of monosaccharide units arranged in different combinations of linkages and asymmetric branching [9]. Only ten monosaccharides namely glucose, galactose, fucose, mannose, xylose, sialic aid, iduronic acid, N-acetylglucosamine (GlcNAc), N-acetylgalactosamine (GalNAc) and glucuronic acid are responsible of creating a complex repertoire of glycans [10]. Most glycans linked to proteins can be classified as N-glycans, attached through nitrogen of Asparagine (Asn) (N-glycosylation), or O-glycans, attached through oxygen of mainly Serine (Ser) or threonine (Thr) (O-glycosylation) [11]. Glycosylation involves co-translational and/or post-translational modifications (PTMs) that are tightly regulated by an enzymatic process that is both substrate and site-specific. Both N- and O-glycosylations have been described in specific cells compartments such as the endoplasmic reticulum, Golgi apparatus and extracellular surface of the plasma membrane [3,11]. In particular, N-glycosylation begins in the endoplasmic reticulum by the synthesis of a lipid-linked oligosaccharide (LLO) precursor, comprising a 14 monosaccharide (Glc_3_Man_9_GlcNAc_2_) sequential assembly on a phosphorylated lipid carrier dolichol. An oligosaccharyltransferase (OST) complex transfers *en bloc* the oligosaccharide part of LLO to the specific site of Asn residue [12]. Differently, O-glycosylation often initiates by the addition of GalNAc to the hydroxyl group of Ser or Thr residues in a polypeptide chain. O-glycans are further extended with the sequential addition of other monosaccharides [13].

O-glycosylation comprises several types of glycans and pathways, where a complex carbohydrate chain may induce stable modifications of substrates. A distinct O-glycosylation occurring independently of endoplasmic reticulum-Golgi apparatus was reported for the first time by Torres and Hart, who described a particular pathway of glycosylation, where a single monosaccharide GlcNAc was linked to residues on the surface of lymphocytes [14]. Further studies demonstrated that this type of glycosylation consisted in O-linked β-N-acetylglucosaminylation (O-GlcNAcylation), where GlcNAc was added on the oxygen of Ser or Thr residues of proteins located in specific cellular compartments including cytoplasmatic membranes, mitochondria and nucleus [9,13,15,16]. O-GlcNAcylation is distinct from all other common forms of protein N- or O-glycosylation, since it occurs exclusively within the nucleus and cytoplasm and it is not further elongated or modified.

**Figure 1:**
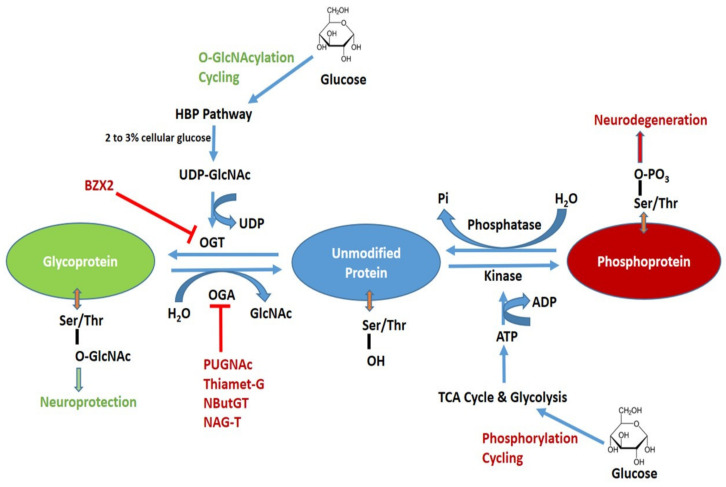
Schematic diagram of O-GlcNAcylation and phosphorylation processes of protein and their possible pharmacological interventions. Hexosamine biosynthetic pathway (HBP) leads to UDP-GlcNAc formation and regulates O-GlcNAcylation. O-GlcNAc transferase (OGT) is inhibited by BZX2 whilst O-GlcNAcase (OGA) is inhibited by PUGNAc, Thiamet-G, NButGT and NAG-T. These OGT and OGA inhibitors are targeted to modulated O-GlcNAc levels in neurodegenerative diseases. Tricarboxylic acid (TCA) and glycolysis cycle regulate protein phosphorylation. Kinase and phosphatase regulate phosphorylation and dephosphorylation, respectively.

Moreover, unlike the complex N- and O-linked glycosylation that is able to induce stable modifications of substrates, O-GlcNAcylation causes a more dynamic PTM and is rapidly reversible in response to various environment stresses [17]. To this regard, O-GlcNAc interplays with other PTMs including O-phosphorylation in a reciprocal manner, which modulates other PTM’s functional interactions with common substrates [17,18,19]. However, not all phosphorylation sites compete with O-GlcNAc sites and vice versa [17]. ATP produced from glycolysis and tricarboxylic acid (TCA) cycle is involved in protein phosphorylation. Kinases and phosphatases drive phosphorylation and dephosphorylation respectively and they are reciprocally regulated by O-GlcNAcylation [20,21]. An increase in O-GlcNAc levels decreased phosphorylation at 280 sites and increased phosphorylation at 148 other sites in NIH 3T3 cultured cells [21]. The complete schematic representation of O-GlcNAc and phosphorylation cycling processes are shown in Figure 1.

More than 4000 proteins have been identified as potential O-GlcNAc candidates and these are involved in all aspects of cellular metabolism [18]. In particular, many proteins in the brain are subjected to glycosylation, including β-amyloid (Aβ) associated protein [22,23], tau [24,25], and neurofilament proteins [25,26]. Dysfunction in the regulation of phosphorylation/glycosylation and glycosylation–deglycosylation processes could contribute to the etiopathology of Alzheimer’s disease (AD) [2].

This review aims to summarize the molecular and cellular mechanisms of glycosylation focusing on specific proteins, which play a crucial role on the onset and progression of AD. In particular, this review will focus more on O-GlcNAcylation, which is highly involved in the dynamic and rapid PTMs of proteins. The understanding mechanisms of glycosylation proteins in this neurodegenerative disorder may open up novel therapeutic strategies. Enhancing our understanding of the mechanisms of protein glycosylation may inform the development of novel therapeutic strategies in AD.

## 2. Biosynthesis and Modulation of O-GlcNA Cylation

Contrary to the complex N- and O-linked glycosylation, which requires several enzymes, O-GlcNAcylation is regulated by the concerted action of only two enzymes: O-GlcNAc transferase (OGT) and O-GlcNAcase (OGA). For a detailed description of OGT and OGA substrate recognition see Joiner et al., 2019 [27]. OGT catalyzes the transfer of GlcNAc onto Ser and Thr residues employing Uridine 5′-diphospho-N-acetylglucosamine (UDP-GlcNAc) as a substrate, whereas OGA is the enzyme that removes O-GlcNAc from proteins [28,29]. OGT is mainly expressed in three predominant isoforms namely short OGT (sOGT), mitochondrial OGT (mOGT) and nucleocytoplasmic OGT (ncOGT). The most studied isoform is ncOGT, the primary product of *ogt* gene, which catalyzes the transfer of O-GlcNAc by an ordered and sequential bi-bi mechanism [30]. OGT mRNA levels are expressed in all tissues, but the highest expression have been reported in the pancreas, brain with reduced levels in other organs. Brain OGT cytosolic activity is ten times higher when compared to other tissues including muscle, heart, liver and adipose tissue [31]. OGA is expressed in two isoforms namely OGA large (OGA-L) and OGA short (OGA-S). OGA-L is localized in both the cytosol and nuclei whilst OGA-S is limited to the nucleus [32]; the latter is relatively six times less active than OGA-L in O-GlcNAc hydrolysis [33,34]. It has been demonstrated that significant OGA expression is mostly restricted to the pancreas, brain and thymus with other organs demonstrating low levels of expression [35].

The hexosamine biosynthetic pathway (HBP) is a branch of the glycolysis that is responsible for the production of UDP-GlcNAc, a key substrate for protein glycosylation. O-GlcNAc is closely regulated by cellular glucose concentrations and 2 to 3% of total glucose is transformed into UDP-GlcNAc [36]. Therefore, O-GlcNAcylation is considered a nutrient sensor and its dependence is not only confined to glucose metabolism but also influenced by amino acid (glutamine), fatty acids (acetyl-CoA) and nucleotide (uridine triphosphate) [37,38]. Thus, fluctuations in nutrition balance could alter its signaling pathway. In fact, it has been demonstrated that hyperglycemic conditions lead to an increase in O-GlcNAc [39], whereas a decreased availability of glucose content induces a considerable reduction in the O-GlcNAc levels [40].

In humans, dysregulation of O-GlcNAcylation occurs in a wide range of diseases, including cancer, diabetes, and neurodegeneration. In the latter context, the pharmacological modulation of the activities of OGT and OGA may be helpful in order to understand their role in neurodegenerative diseases. OGT and OGA inhibitors have been identified, which are able to modulate the glycosylation are listed in Table 1.

## 3. Glycosylation in Alzheimer’s Disease

Alzheimer’s disease (AD) is a neurodegenerative disorder characterized by a progressive decline in memory, attention and language, and histopathologically the deposition of extracellular Aβ plaques and intracellular neurofibrillary tangles (NFTs) are considered the two iconic neuropathological hallmarks of AD at post-mortem [49,50,51,52,53].

Besides Aβ plaques and NFTs, additional molecular events are also related with the progression of the disease and include the increase in the reactive oxygen species (ROS) production, mitochondrial dysfunction, inflammation and a decrease in cerebral glucose uptake/utilization [3,51,54,55]. To the latter regard, it is widely accepted that alterations in brain structure and function precede the signs and symptoms of the disease by many years. During normal aging, glucose uptake in the brain is decreased and this decline is strongly accelerated in Alzheimer’s patients. In this context, several 2-[^18^F]fluoro-2-deoxy-D-glucose (FDG) PET studies have shown that metabolic reductions in patients occur decades before onset of AD symptoms, suggesting that metabolic deficits may be a defining upstream event in at least some late-onset AD cases [56]. These results were also confirmed in other mouse models of AD (e.g., APP/PS1 and 3 × Tg-AD mice), where reduced peripheral glucose tolerance occurs prior to AD-related neuropathology or cognitive decline [57,58,59,60]. The neurons have energy requirements and high metabolic rates and as such, their functionality is directly dependent on glucose supply and utilization, and, therefore, interruptions of the glucose supply make neurons vulnerable to damage. However, the molecular mechanisms and crosstalk between the different signaling pathways involved in the regulation of glucose metabolism and cognitive improvements, or the mechanisms by which these pathways are deregulated in AD, have not been completely identified. To this regard, more recently it has been demonstrated that Wnt signaling plays a crucial role in regulating glucose metabolism in the brain, and this pathway is downregulated in AD [61,62]. In particular, Wnt signaling promotes glucose uptake and utilization in cultured hippocampal neurons and, more interestingly, ameliorates cognitive decline in APPswe/PS1dE9 transgenic mice model AD by enhancing glucose metabolism [62,63]. Therefore, it might be promising to target Wnt pathways for neuroprotection in AD. Wnt pathway is sensitive to alterations in the glycosylation state of a cell and acts as a nutritional sensor in order to couple growth/proliferation with its metabolic status [64]. In particular, UDP-GlcNAc pathway specifically regulates Wnt signaling possibly through the global changes on the glycosylation state of Wnt receptor proteins [65].

Alteration in glucose metabolism seems to lower the UDP-GlcNAc through HBP, which alleviates, in turn, the O-GlcNAc formation [25,66]. The involvement of glucose metabolism in AD has also been supported by the observation that type II diabetes mellitus (T2DM) is a risk factor for, and impaired glucose metabolism correlates with dementia severity in AD [67,68,69,70]. Moreover, it has been reported that the expression levels of glucose transporter 1 (GLUT1) and GLUT3 acting as glucose transporters were significantly decreased in the brain of AD patients and streptozotocin-treated rats together with O-GlcNAcylation, whereas the phosphorylation of tau and neurofilaments were increased [25,71]. Among others, β-amyloid precursor protein (APP), secretases (α-, β- and γ-secretases), neprilysin, tau and other components of the neuron cytoskeleton are candidates for O-GlcNAcylation.

APP is an integral membrane glycoprotein and exists in both mannose-capped immature oligomannose N-glycans and mature complex N-glycans [72,73]. The APP intracellular maturation process, the translocation from the Golgi to the cell membrane and metabolism are driven by glycosylation, sulfation, and phosphorylation [73]. In this regard, the inhibition of N-glycosylation with the mannosidase inhibitors swainsonine or deoxymannojrimycin altered the subcellular translocation of APP with its retention in the Golgi, indicating that changes in APP N-glycosylation state could affect the processing pathway of this protein by determining its transport from the Golgi to the cell membrane [74]. N- glycosylation of APP is required for extracellular secretion in vitro [75]. Sialylation of APP N-glycans could drive the proteolytic cleavage of APP towards the amyloidogenic-processing pathway operated by two proteases, β-site APP cleaving enzyme-1 (BACE1) and γ-secretase complex, thereby playing a crucial role in the development of AD [72,73,76,77,78]. O-GlcNAcylation enhances non-amyloidogenic processing resulting in neuroprotective effects [35]. O-GlcNAcylation of APP at Thr 576 is the main regulator of Aβ trafficking and processing, making this residue a possible drug target in AD [79].

α-secretase cleaves APP within the Aβ sequence and, together with γ-secretase, generates a soluble APP intracellular domain (AICD) and a secreted sAPPα fragment, which has been reported to have neuroprotective properties [80]. In this regard, increased levels of O-GlcNAcylated APP using PUGNAc augmented α-secretase activity together with an increased level of sAPPα and a reduction of Aβ40 in SH-SY5Y neuroblastoma cells [42]. α-secretase is a member of ‘a disintegrin and metalloprotease’ (ADAM) family and, in particular, ADAM10 is the constitutive α-secretase in primary neurons [81]. Interestingly, inactivation of ADAM10 in mice (*Adam10^−/−^*) resulted in complete suppression of α-cleavage in primary neurons enhancing Aβ42 levels [82,83]. ADAM10 is a type I transmembrane glycoprotein that cleaves several plasma membrane proteins and like other ADAMs is N-glycosylated [84]. ADAM10 has four potential N-glycosylation sites (N267, N278, N439 and N551), which contain high-mannose or complex-type glycans [84]. Individual N-glycosylation site mutants were constructed, and results demonstrated that each of the N-glycosylation sites from ADAM10 is required for full-enzyme activity [84]. Further investigation will be required elucidating the precise functional role played by ADAM10 N-glycosylation in AD.

BACE1, (also known as β-secretase) cleaves APP and is a crucial protease for the generation of neurotoxic peptide Aβ42. BACE1 is an aspartic protease with four N-glycosylation sites [85]. Site-directed mutagenesis eliminating all BACE1 N-glycans resulted in very slow intracellular maturation suggesting that N-glycans are important in folding and maturation of BACE1 [86]. In the mouse brain, BACE1 contains N-glycans, which are further modified by β1,4-N-acetylglucosaminyltransferase III (GnT-III) that is encoded by the *Mgat3* gene [87]. The product of GnT-III is referred to as a bisecting GlcNAc linkage, where GnT-III catalyzes the addition of GlcNAc to a mannose residue linked through a β1,4-linkage [88]. The introduction of the bisecting GlcNAc prevents further processing because this structure cannot act as a substrate for other glycosyltransferases [87]. Post-mortem evaluation of AD patients revealed that the level of bisecting GlcNAc on BACE1 is elevated with disease progression, suggesting that this abnormal change in BACE1 glycosylation may be involved in AD pathogenesis by modulating β-cleavage of APP [72,87]. It has been well established that oxidative stress plays a crucial role in the neurodegeneration and, more specifically, it induces an up-regulation of BACE1 expression leading to enhance Aβ42 accumulation [53,89]. Kizuka and colleagues demonstrated that bisecting GlcNAc could modulate the expression of BACE1 induced by oxidative stress [85]. In particular, Kizuka and colleagues demonstrated that the absence of bisecting GlcNAc drives BACE1 towards the lysosomal degradation of BACE1, which, in turn, leads to a reduction in Aβ42 deposition. H_2_O_2_-treated *Mgat3^−^^/^^−^* cells showed a rapid degradation of BACE1 that was abolished by inhibiting lysosomal hydrolases by chloroquine [85]. These results demonstrated that oxidative stress-induced high BACE1 levels were significantly reduced in *Mgat3^−/−^* cells due to accelerated lysosomal degradation. GnT-III-deficient (*Mgat3^−^^/^^−^*) mice, which do not demonstrate obvious gross phenotypic abnormalities, were crossed with APP23 transgenic mouse model of AD, which expresses human APP (hAPP) [87,90]. Interestingly, in these *hAPP/Mgat3^−/−^* mice, the authors found significant reductions of both Aβ40 and Aβ42 levels in an age-dependent manner, which was accompanied by a markedly decreased number of Aβ plaques and an amelioration of the cognitive impairment [87]. These latter results suggest that inhibiting GnT-III may be a promising therapeutic strategy with an advantageous profile of adverse drug reactions compared to BACE1 inhibitors. In this regard, preclinical studies demonstrated that BACE1-deficient mice displayed severe abnormalities including schizophrenia-like behaviors, hypomyelination and early lethality, which suggests that BACE1 inhibitors may exert alarming adverse effects [91,92]. This is further confirmed by the high failure and premature termination rate of recent clinical trials of drugs targeting BACE1, many of which were at an advanced stage [93].

γ–secretase catalyzes the second proteolytic cleavage of APP within its transmembrane region after the proteolytic interventions of α–secretase or BACE1 [52]. γ-secretase is a protease complex and consists of four essential subunits: presenilin (PSEN) (including PSEN1 and 2), nicastrin, anterior pharynx-defective 1 (Aph-1) and presenilin enhancer 2 (Pen-2) [94,95,96,97,98]. γ-secretase is involved in a vast range of crucial biological activities and, besides APP, it cleaves the Notch intracellular domain (NICD), which is able to translocate into the nucleus and regulate gene expression controlling cell proliferation, survival, positioning and differentiation [94,95]. Therefore, when γ-secretase was considered as a potential key target for the development of AD-modifying therapeutics, its genetic and pharmacological manipulation revealed severe developmental defects and serious safety issues [99,100]. Nicastrin is the only glycosylated subunit having 16 possible N-glycosylation sites and the mature protein contains a mixture of high-mannose and complex oligosaccharide side chains generated during trafficking through the Golgi apparatus [101]. PSEN1/2 play a crucial role in the glycosylation process and maturation of nicastrin, as demonstrated in *Psen1/2* double-knockout cells, where the mature form of nicastrin is completely lost [97]. Surprisingly, both Aβ and NICD productions were not affected by pharmacological inhibition of mannosidase-I, suggesting that nicastrin glycosylation is not required for γ-secretase activity. However, it is required for the maturation of the protein before its incorporation into the γ-secretase complex or its cell-surface localization [101]. To further understand the relationship between the O-GlcNAcylation of γ-secretase and AD neuropathology, a mouse model of AD carrying 5 × FAD genes was treated with NButGT, a specific OGA inhibitor [102]. NButGT-treated 5 × FAD mice showed reduced levels of Aβ42 and Aβ40 peptides leading to a reduction in amyloid plaque formation, attenuated neuroinflammation and improvements in cognition. Interestingly, NButGT treatment decreased γ-secretase activity without affecting the α- or β-secretase activity [102]. More research will be needed in the near future to better understand the structure-function relationship of γ-secretase in order to identify more specific inhibitors, which selectively modulate Aβ production without affecting Notch cleavage.

**Figure 2:**
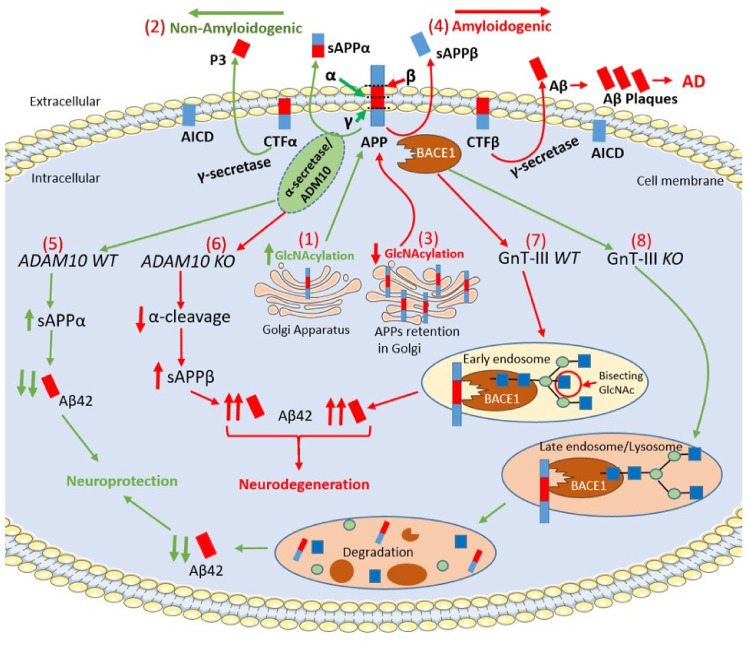
Schematic representation of APP intracellular maturation and its cleavage by secretases. (1) β-Amyloid precursor protein (APP) undergoes glycosylation in the Golgi apparatus and, then, translocates to cell membrane for secretase cleavage (2) The increase of APP glycosylation drives toward the non-amyloidogenic pathway. α-secretase cleaves APP within the β-Amyloid (Aβ) sequence producing the C-terminal fragment α (CTFα) and soluble N-terminal fragment α (sAPPα), which has neuroprotective properties. Afterwards, γ-secretase cleaves CTFα into N-terminal fragment (P3) and a soluble APP intracellular domain (AICD). (3) The pharmacological inhibition of APP glycosylation retains the protein in the Golgi apparatus preventing its transport to cell membrane. The reduction of the glycosylation process drives the amyloidogenic pathway. (4) In the amyloidogenic pathway, β-secretase cleaves APP producing soluble N-terminal fragment β (sAPPβ) and membrane bound C-terminal fragment β (CTFβ). Then, γ-secretase processes CTFβ into AICD and Aβ42, which forms extracellular Aβ plaques. (5) α-secretase is a member of ‘a disintegrin and metalloprotease’ (ADAM) family and, in particular, ADAM10 cleaves APP increasing the levels of sAPPα leading to neuroprotection. (6) The genetic deletion of ADAM10 suppresses the α-cleavage of APP, decreases in sAPPα levels and increases Aβ42 formation in ADAM10 knockout (KO) mice (*Adam10^−/−^*). (7) β-site APP cleaving enzyme-1 (BACE1) contains N-glycans, which are further modified by a glycosyltransferases, namely β1,4-N-acetylglucosaminyltransferase (GnT-III) that is encoded by the Mgat3 gene. In GnT-III WT mice (*Mgat3+/+* mice), BACE1 localizes in early endosomes and is modified by GnT-III to bear bisecting GlcNAc generating Aβ42 elevation. (8) In contrast, in GnT-III knockout (KO) mice (Mgat^−/−^ mice), BACE1 is relocated to late endosomes/lysosomes where BACE1 is degraded leading to a decrease of Aβ42 levels. (Red/green up arrows: Increase; Red/green down arrows: Decrease).

Neprilysin is a zinc metallopeptidase identified in the brain and it is the major rate-limiting enzyme involved in Aβ42 degradation [103,104]. Brain regions with high plaque deposition, including the hippocampus and temporal gyrus, were inversely correlated with a selective reduction in neprilysin expression, suggesting that downregulation of neprelysin levels could promote Aβ deposition in the brain [103,104]. The expression, stability and enzymatic activity of neprilysin were affected by N-glycosylation and its activity completely lost upon N-glycosylation removal induced by glycopeptidase F treatment. Furthermore, site-directed mutagenesis of the N-glycosylation sites in neprilysin reveals that an N-glycan at Asn628 is crucial not only for neprilysin expression but also modulates its activity that affects Aβ clearance [103,104,105]. All the above-mentioned mechanisms are depicted in Figure 2.

Microtubule-associated protein (MAP) tau is a phosphoprotein and promotes the assembly and disassembly of microtubules by phosphorylation at Ser or Thr residues [106]. Besides phosphorylation, tau is extensively modified with O-GlcNAc at the same sites [107]. In fact, tau protein exhibits multiple O-GlcNAc bearing sites in the brain and an increase in tau-O-GlcNAc reduces tau phosphorylation [107]. Tau-O-GlcNAc is neuroprotective whilst its hyperphosphorylation leads to the formation of the toxic NFTs observed in AD human and mouse brain [106]. A triple transgenic murine model of AD (3 × Tg-AD), which develop age-dependent and region-specific Aβ and tau aggregations that closely mimic the disease progression seen in humans, represents a valuable model for the study of O-GlcNAcylation [23,108,109,110,111,112,113]. In this regard, 12-month-old 3 × Tg-AD mice exhibited reduced levels of tau O-GlcNAcylation, which is consistent with tau hyperphosphorylation at Ser396 and Thr205 in the hippocampus [22]. The reduction of O-GlcNAcylation was region-specific and was not associated with changes in expression or activity of O GlcNAcylation regulatory enzymes. In fact, the expression of OGT, OGA, and glutamine-fructose-6-phosphate amido transferase (GFAT), the HBP rate-limiting enzyme, remained unaffected in the hippocampus, hinting that the reductions in O-GlcNAcylation may be due to defective glucose metabolism [22]. This hypothesis is supported by the clinical observations where AD patients displayed hypo-O-GlcNAc and hyperphosphorylation of tau that was accompanied by impaired glucose metabolism [43]. Thus, de-glycosylated tau becomes a substrate for kinases leading to tau phosphorylation. These data suggest that tau-O-GlcNAc and tau phosphorylation are inversely related and have opposite effects, which suggests that O-GlcNAc is a possible novel therapeutic target in AD. Tramutola and colleagues reported an increase of OGA enzyme activity in 3 × Tg-AD, coupled with the decrease of total O-GlcNAcylation levels, which could translate as an increased removal of GlcNAc residues from proteins [23]. The pharmacological inhibition of OGA with Thiamet-G in the JNPL3 tauopathy mouse model resulted in increased tau O-GlcNAc and decreased neuronal cell loss and tau aggregate formation [114,115]. Similarly, targeting tau-O-GlcNAc with OGA inhibitor PUGNAc in PC12 cells has been shown to decrease tau phosphorylation and raise the O-GlcNAc levels [43]. Each inhibitor activates different downstream pathways. For example, Thiamet-G stimulates autophagy through a mammalian target of rapamycin-independent pathway in neuroblastoma N2a cells, primary rat neurons and mouse brain, which helps the brain to combat the accumulation of toxic protein species [115]. Besides the pharmacological inhibition of OGA, BZX2, an OGT inhibitor, leads to a 2-fold increase in tau phosphorylation at Ser199 and 1.5-fold increase at Ser396 inducing tau aggregation [41].

Besides tau, altered PTM of other components of the neuron cytoskeleton, which contribute to NFTs formation during AD, have been reported [23]. In addition to tau hyperphosphorylation, components of microtubule structure, α- and β-tubulin, and components of neurofilaments are known to be impaired in AD [116,117]. By proteomic analysis, it has been demonstrated that 12-month-old 3 × Tg-AD mice exhibited reduced O-GlcNAcylation of β-tubulin and neurofilament light chain that comprises the intermediate filaments structure [23]. Therefore, decreased O-GlcNAcylation and increased phosphorylation levels of these microtubule structures may play a detrimental role for NFT formation, confirming the existence of reciprocal regulation between O-GlcNAcylation and phosphorylation.

## 4. Conclusions

The present review provides evidence that the alterations of the glycosylation process play an important role in the aetiology and pathogenesis of AD. Despite some conflicting results, preclinical and clinical studies point to an impairment/downregulation of O-GlcNAcylation during the progression of AD. Therefore, this review supports the idea that the glucose metabolic pathway may represent an alternative therapeutic option for targeting AD. In this perspective, the pharmacological modulation of O-GlcNAcylation levels may represent a ‘sweet approach’ to treat AD targeting new mechanisms independent of the amyloid cascade and with comparable impacts in familial and sporadic AD.

## Figures and Tables

**Table 1:** Effects of OGT and OGA Inhibitors.

Name	Subjects	Effects	References
OGT Inhibitor:			
**BZX2**	Tau-BiFC cells	↑ tau phosphorylation at Ser199 and Ser396	[41]
		↑ tau aggregation	
OGA Inhibitors:			
O-(2-acetamido-2-deoxy-d-glucopyranosylidene)amino-N-phenylcarbamate	SH-SY5Y cells	↑ O-GlcNAcylation, ↑sAPPα,	[42]
(PUGNAc)	PC12 cells	↓ Aβ levels	[43]
		↓ Phosphorylation level of tau at Ser-199, Ser-202, Thr-205, Thr-212, Ser-214, Ser-262, and Ser396	
1,2-dideoxy-2′-methyl-alpha-d-glucopyranoso[2,1-d]-Delta2′-thiazoline	Human O-GlcNAcase	Highly selective competitive OGA inhibitor	[44]
(NAG-thiazoline)	NIH 3T3 cells	↑ Global GlcNAcylation levels	[21]
1,2-dideoxy-2′-propyl-alpha-D-glucopyranoso-[2,1-D]-delta2′-thiazoline	Sprague-Dawley rats	↑ O-GlcNAc levels	[45]
(NButGT)	C57BL/6J mice	No alteration in glucose tolerance and insulin signaling pathways	[46]
		No insulin resistance	
3ar,5r,6s,7r,7ar)-2-(Ethylamino)-5-(Hydroxymethyl)-5,6,7,7a-Tetrahydro-3ah-Pyrano[3,2-D][1,3]thiazole-6,7-Diol	TAPP mice	↑ Global GlcNAcylation levels	[47]
(Thiamet-G)		Improves performance in the Morris water maze (MWM) test	
		↓ Aβ levels	
N-(5-(((2S,4S)-2-methyl-4-(6-fluoropyridin-2-yloxy)piperidin-1-yl)methyl)thiazol-2-yl)acetamide (LSN3316612)	Rhesus monkey	↑ O-GlcNAc levels	[48]
	Oga knock-out mice	↓ Tau phosphorylation	

Note: ↑: Increase; ↓: Decrease.

## Abbreviations

AD	Alzheimer’s disease
ADAM	A disintegrin and metalloprotease
AICD	APP intracellular domain
APP	β-amyloid precursor protein
Aph-1	Anterior pharynx-defective 1
Asn	Asparagine
Aβ	β-amyloid
BACE1	β-site APP cleaving enzyme-1
FDG	Fluoro-2-deoxy-D-glucose
GalNAc	N-acetylgalactosamine
GFAT	Glutamine-fructose-6-phosphate amidotransferase
GlcNAc	N-acetylglucosamine
GLUT	Glucose transporter
GnT-III	β1,4-N-acetylglucosaminyltransferase III
HBP	Hexosamine biosynthetic pathway
hAPP	human APP
LLO	Lipid-linked oligosaccharide
MAP	Microtubule-associated protein
Mgat3^−/−^	GnT-III-deficient
mOGT	Mitochondrial OGT
ncOGT	Nucleocytoplasmic OGT
NFTs	Neurofibrillary tangles
NICD	Notch intracellular domain
OGA	O-GlcNAcase
OGA-L	OGA Large
OGA-S	OGA short
O-GlcNAcylation	O-linked β-N-acetylglucosaminylation
OGT	O-GlcNAc transferase
OST	Oligosaccharyltransferase
Pen-2	Presenilin enhancer 2
PET	Positron emission tomography
PSEN	Presenilin
PTMs	Post-translational modifications
ROS	Reactive oxygen species
Ser	Serine
sOGT	short OGT
TCA	Tricarboxylic acid
Thr	Threonine
T2DM	Type II diabetes mellitus
UDP-GlcNAc	Uridine 5′-diphospho-N-acetylglucosamine
3 × Tg-AD	Triple transgenic murine model of AD
